# Correlation between image characteristics and pathologic findings in non small cell lung cancer patients after anatomic resection

**DOI:** 10.1371/journal.pone.0206386

**Published:** 2018-10-31

**Authors:** Jui-Ying Fu, Yung-Liang Wan, Tzu-Yen Huang, Ching-Feng Wu, Yun-Hen Liu, Ming-Ju Hsieh, Yi-Cheng Wu, Ching-Yang Wu

**Affiliations:** 1 Chang Gung University, Taoyuan, Taiwan; 2 Division of Pulmonary and Critical Care, Department of Internal Medicine, Chang Gung Memorial Hospital at Linkou, Taoyuan, Taiwan; 3 Department of Medical Imaging and Intervention, Chang Gung Memorial Hospital at Linkou, Taoyuan, Taiwan; 4 Division of Thoracic and Cardiovascular Surgery, Department of Surgery, Chang Gung Memorial Hospital at Keelung, Keelung City, Taiwan; 5 Division of Thoracic and Cardiovascular Surgery, Department of Surgery, Chang Gung Memorial Hospital at Linkou, Taoyuan, Taiwan; Baylor College of Medicine, UNITED STATES

## Abstract

**Introduction:**

Tumor characteristics in computed tomography (CT) are correlated to pathologic presentation and survival. However, most studies have been based on thin slice thickness CT while lymph node metastatic pattern has remained unclear. The aim of this study was to analyze the correlation between image characteristics under 5 mm slice thickness and pathology findings in non small lung cancer patients who have received curative resection.

**Materials and methods:**

From January 2010 to May 2014, 440 patients who underwent curative resection were included and medical records were reviewed retrospectively. The tumor size and consolidation tumor ratio were simultaneously evaluated and measured by a physician, a thoracic surgeon, and a radiologist. The correlation between image and pathology characteristics and its survival impact was analyzed.

**Results:**

Tumor sizes, as measured by CT and by pathologic measurement were highly coincident. (p < 0.001) GGO predominant lesions were correlated to well-differentiated adenocarcinoma, (p< 0.001), and less tumor necrosis (p<0.0001), lymphocyte infiltration (p = 0.0042) and tumor purity (p <0.0001). In addition, less risk of visceral pleura (p < 0.0001) and angiolymphatic invasion, and fewer metastases to N1 lymph node (p = 0.004) involvement were identified. No lymph node metastasis (0/12) was identified in sub-centimeter pure GGO lesion. The consolidation tumor ratio could be used to differentiate patients’ survival and excellent 5-year overall survival was identified in pure GGO lesion cases.

**Conclusion:**

No lymph node metastasis was identified in sub-centimeter pure GGO lesion. The consolidation tumor ratio could be used to differentiate patients’ disease status and overall survival, while excellent 5-year overall survival was identified in cases with pure GGO lesion.

## Introduction

Lung cancer is the leading cause of cancer death worldwide. [1–6] According to data from the Ministry of Health and Welfare of Taiwan, the death rate due to lung cancer in 2015 was 199.6 per 100,000 population. [7] For patients with resectable disease, the cumulative disease free and overall survival rate decreases as the cancer staging increases. [5,8,9] For those with un-resectable disease, ie. stage IIIb or IV, the prognosis is grave despite aggressive treatment. [8–18] Identification of early stage lung cancer is crucial for better survival. Therefore, low dose computed tomography (CT) screening for patients at risk has been proposed [19,20] and more lesions are being identified from the image survey. Hence, the ability to distinguish malignant lesions from other non-specific findings is crucial for clinical practitioners. The identified lesions could be ground glass opacity (GGO) or solid lesions, whereby ground glass opacity (GGO) lesions could be either malignant or benign, [21–22] with less invasive pathologic characteristics and better overall survival. [23–25] Solid lesions have higher lymph node involvement and worse overall survival. [23,26] In addition, tumor size and components, ie. consolidation-tumor ratio, have also been correlated to patients’ survival. [27–31] Based on these findings, Kudo Y et al. demonstrated the relationship between CT characteristics and pathologic findings.[32] However, there has been no consensus on CT slice thickness and most reported findings are based on 1 mm slice thickness which is not always available in daily clinical practice. As the slice thickness decreases, the radiation dose increases. which not only increases lesion detection rate but also image noise. [33,34] In addition, different tumor component presentations could be encountered in the same tumor under different image settings. These could limit the clinical applicability of these studies.

For clinical practitioners, it is crucial to decide what a given CT image may imply in order to make the best recommendation, such as undergoing regular surveillance or commencing surgical resection. In daily clinical practice, most patients present at an out-patient clinic with no other imaging tools besides CT obtained with 5mm slice thickness. Patients with suspected lung lesions are worried about the possibility of lung cancer and are eager to know the survival impact if pulmonary malignancy is confirmed. Hence the question arises how to correctly interpret the characteristics of CT findings. In order to solve this problem and provide quantified data for decision making, we analyzed radiologic and pathologic findings of non small cell lung cancer patients who received anatomic resection and mediastinal lymph node dissection. We not only tried to analyze the relationship between radiological and pathological characteristics but further identified the survival impact, when correlated to tomographic characteristics. Combining our result and currently available guidelines, we are able to provide more precise recommendations for patients with indeterminate lung lesions.

## Materials and methods

### Patient selection and data collection

**Fig 1:**
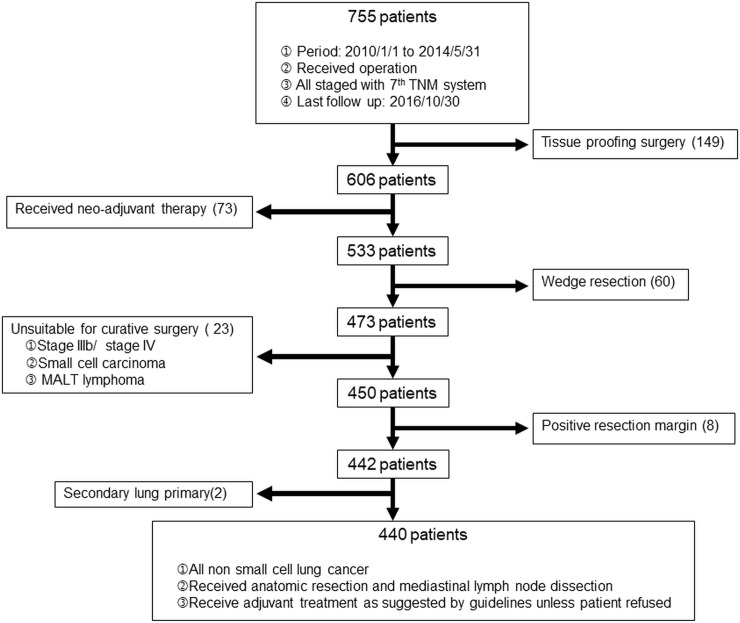
Exclusion algorithm.

From January 2010 to May 2014, 755 non small cell lung cancer patients who underwent surgery were enrolled in this study. The exclusion algorithm is shown in Fig 1. To analyze the correlation between CT image and pathologic characteristics, 440 patients who underwent anatomic resection and mediastinal lymph node dissection were included. Medical records, including clinical and pathologic data were reviewed retrospectively. This study was approved by the Institutional Review Board of Chang Gung Medical Foundation under the IRB number 103-5361B.

### Patient pre-operation evaluation

Pre-operation evaluation consisted of two parts; the anatomic study and the cardiopulmonary reserve survey. For patients with confirmed diagnosis, complete pre-operation anatomic survey, including chest CT, positron emission-computed tomography (PET-CT) and brain magnetic resonance imaging (MRI) or CT were arranged to exclude possible distant metastases. Additional procedures, such as endobronchial ultrasonography or neck lymph node excision biopsy were done for possible N3 lesions. For those without preoperatively confirmed diagnosis, chest CT was done for anatomic survey. PET-CT and brain MRI were arranged to exclude extrapulmonary lesions. All patients received pre-operative cardiopulmonary reserve evaluation. Mandatory preoperative spirometry was arranged to confirm adequate pulmonary reserve. For patients with multiple comorbidities, echo cardiography was done and an anesthesiologist was consulted for pre-operative risk evaluation. Only patients with resectable disease and adequate cardiopulmonary reserve underwent anatomic resection and mediastinal lymph node dissection.

### Operation and post-operation adjuvant therapy

All patients enrolled in this study had received anatomic resection, such as lobectomy, bi-lobectomy or pneumonectomy, and mediastinal lymph node dissection. All operations were intended to be done by video-assisted thoraco-scopic surgery (VATS) technique. Only patients with huge mass, severe adhesion, or vessel injuries, were shifted to open thoracotomy to complete the intended curative surgery. Post-operative adjuvant therapy was given according to corresponding pathologic stage and recommendations of the NCCN guideline. Post-operative adjuvant therapies were given to all patients except stage Ia patients.

### Surveillance program and definition of timetable

Patients were followed up in the outpatient department (OPD) every 3 months. Computed tomography from the lower neck to the upper abdomen was used as the surveillance imaging tool and arranged in 3 or 6 month-intervals. The last follow up date was defined as the last date of return to OPD. The relapse date was defined as the date of disease relapse confirmation by image evidence or pathology confirmation after renewed biopsy. The expiry date was defined as patient death or date of critical discharge against advice. The disease-free survival (DFS) was defined as lasting from diagnostic date (1^st^ pathology or image confirmation date) to relapse date. The overall survival (OS) was defined as lasting from diagnostic date (1^st^ pathology or image confirmation date) to last OPD or expiry date.

### Definition of CT characteristics re-evaluation

**Fig 2:**
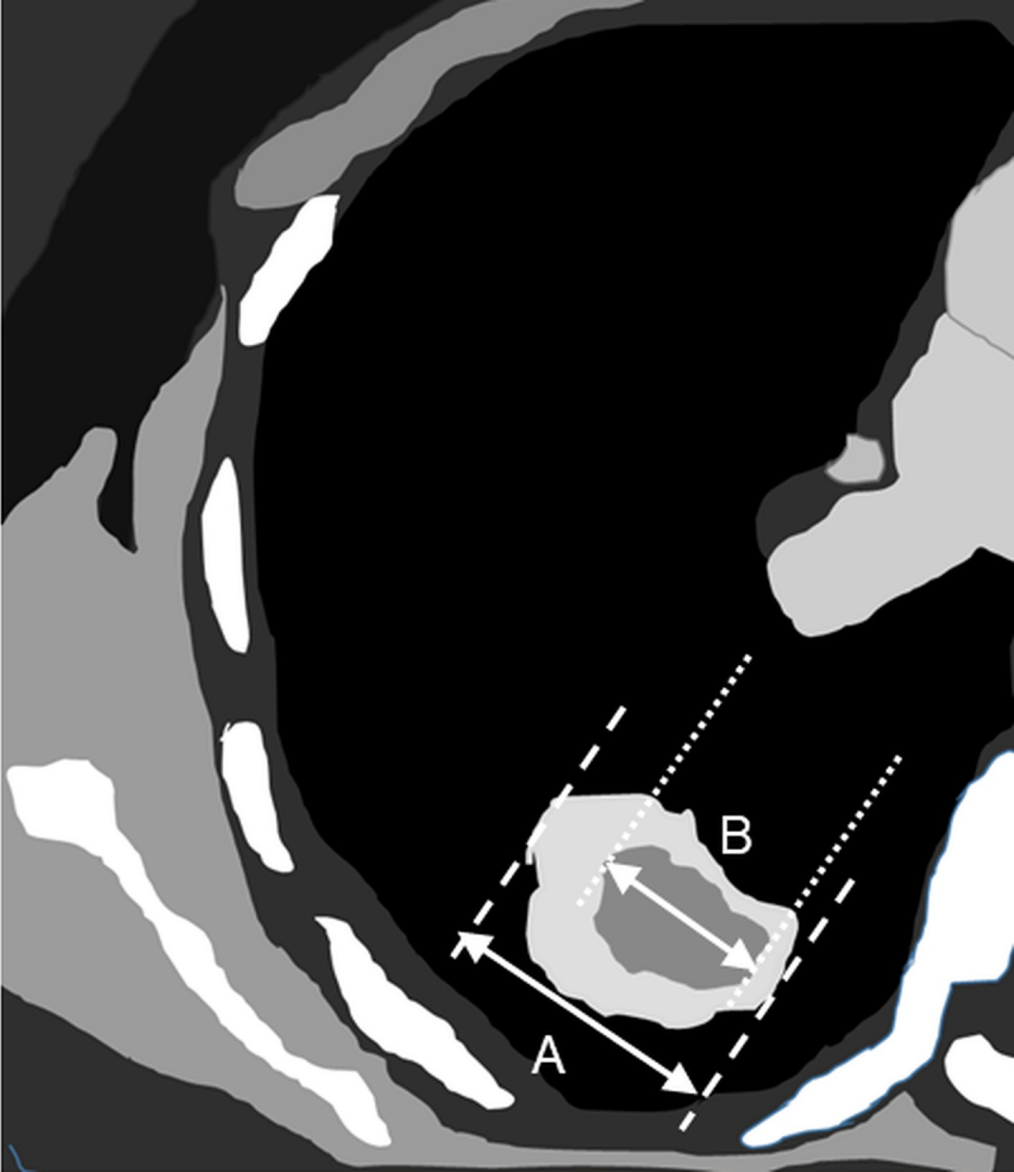
Definition of consolidation tumor ratio (B/A). A: Maximal diameter of the consolidation part of the tumor was measured in CT cross section under lung window view. B: Maximal diameter of the consolidation part of the tumor was measured in CT cross section under mediastinal window.

**Fig 3:**
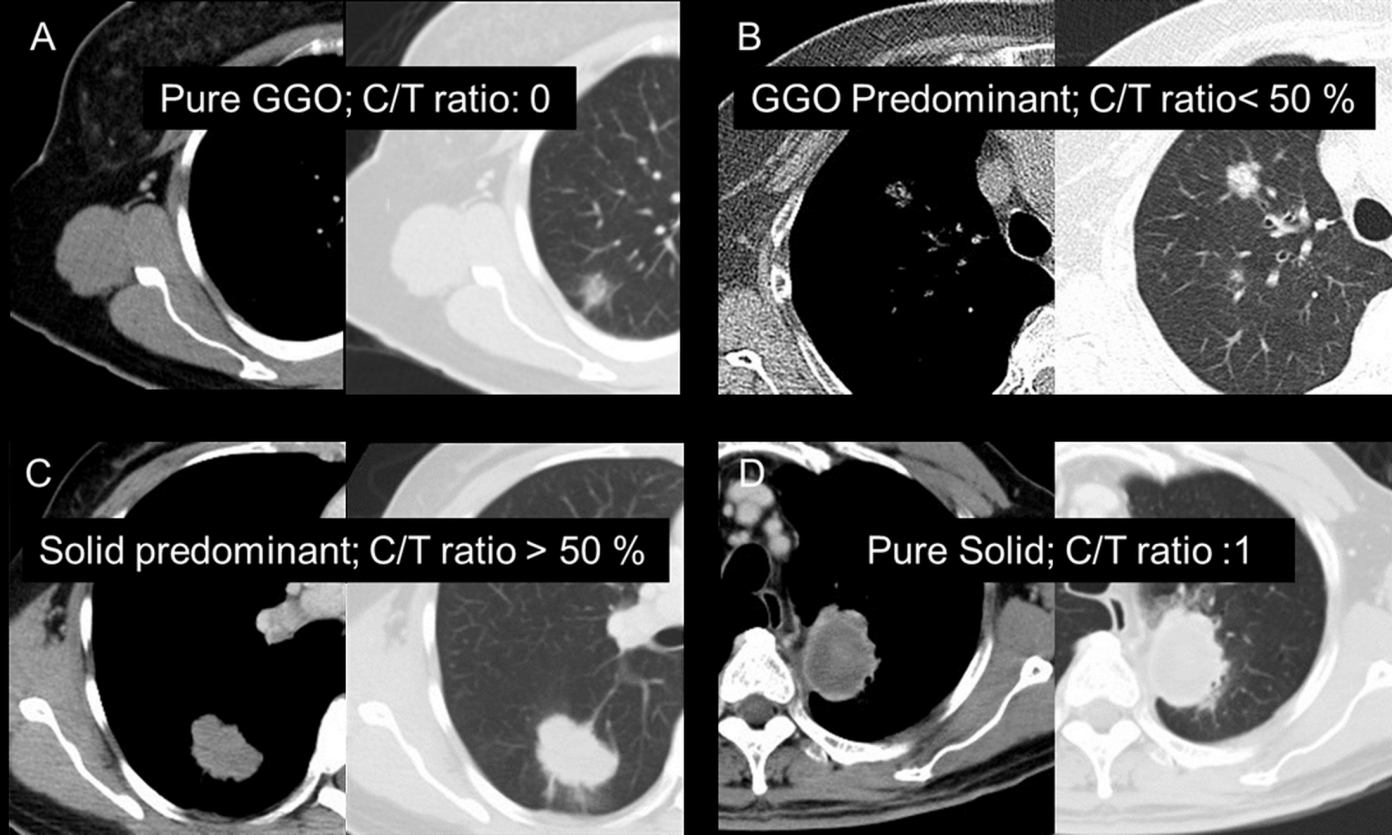
Classifications of tumor component. A. Pure ground glass opacity (GGO) B. Ground glass predominant C. Solid predominant D. Pure solid.

Chest CT's of all enrolled patients were of 5 mm slice thickness. The images were reviewed by a physician, a surgeon, and a radiologist at the same time to eliminate differences between clinical practitioners and radiologists. Prior measurement, and the target image in the lung window were selected by all specialists. Only lesions which presented with maximal tumor diameter at their long axis in the lung window were selected and measured. Tumor size was defined as the maximal diameter of the tumor (A), as measured in CT cross section under lung window view. The maximal diameter of the consolidation part of the tumor (B) was also measured in CT cross section in mediastinal window. The consolidation-tumor ratio (C/T ratio) was defined as B/A and shown in Fig 2. There were four different types of clinical presentations, including pure ground glass opacity (GGO), ground glass predominant (solid part <50%), solid predominant (solid part > 50%) and pure solid. The actual CT presentation is shown in Fig 3.

### Statistics

All collected clinico-pathologic factors were evaluated by univariate analysis. Categorical variables were compared using chi-squared tests and Fisher's exact test while continuous variables were compared using the two sample t-test. Pearson correlation was the correlation statistic used to measure the degree of relationship between linearly related variables. A linear model was used to describe the relationship between a dependent variable and independent variables (three or more groups). Cox proportional hazard model was used for multivariate analysis. Survival status was represented with a Kaplan–Meier curve and compared using the log-rank test. A p-value < 0.05 was considered statistically significant. All analyses were performed using SAS, version 9 (SAS Institute, Cary, NC).

## Results

### Descriptive data for non small cell lung cancer patients who received anatomic resection

The mean age was 62.06±11.4 years, and 51.59% of the patients were male. The mean tumor size as measured on CT was 3.35 ± 1.82 cm. The mean C/T ratio was 0.72 ± 0.28. The major tumor component presentation revealed on CT was solid predominant (269 patients, 61.7%). 92.5% of patients received video assisted thoracoscopic anatomic resection and mediastinal lymph node dissection. The rate of conversion to thoracoscopic surgery was 6.36% (28/440). Total time in the operating room was 208.14 ± 57.14 minutes and the mean blood loss was 115.4 ml. The mean hospital stay was 13.27 days and mean intensive care unit stay was 0.78 days. There was no in-hospital mortality. Of the pathologic findings, adenocarcinoma was the major cell type (335/440, 76.14%) and the total dissected mediastinal nodes were 21.22 ± 11.03. In this cohort, patients with stage Ia and Ib constituted the majority (Stage Ia:148, 33.86%; Stage Ib: 141, 32.06%) (Table 1).

**Table 1:** Descriptive data for non small cell lung cancer patients who received anatomic resection.

Variables	(Mean ± SD)/ Mean (%)	Variables	(Mean ± SD)/ Mean (%)
Age¤	62.06±11.40	Cell type	
Gender Female	213 (48.41)	Adenocarcinoma	335 (76.14)
Body height	160.47±11.13	Squamous cell carcinoma	44 (10.00)
Body weight	63.08±11.13	Mucinous Adenocarcinoma	10 (2.27)
CT finding Tumor size	3.35±1.82	Lympho-epithelioma-like carcinoma	10 (2.27)
CT finding Tumor ratio	0.72±0.28	Other	41 (9.32)
CT tumor component		Tumor purity	0.41±0.38
Pure GGO	33 (7.57)		
GGO predominant	44 (10.09)		
Solid predominant	269 (61.70)		
Pure Solid	90 (20.64)		
Other^1^	4		
VATS/Thoracotomy		Differentiated grade	
Thoracotom	7 (1.6)	G1	201 (45.68)
VATS→Thoracotomy^2^	28 (6.36)	G2	138 (31.36)
VATS	405 (92.05)	G3	57 (12.95)
Total staplers	7.97±2.82	G4	16 (3.64)
Blood Loss (ml)	115.40±236.62	N/A	28 (6.36)
OR time (min)	208.14±57.14	Lymph node status	
Post OP days	8.46±8.56	Total LN No.	21.22±11.03
Postoperative in-hospital stay	13.27±10.77	Metastatic N1 LN No.	0.42±1.10
ICU stay (days)	0.78±6.17	Metastatic N2 LN No.	0.40±1.31
In hospital mortality	0	Pathologic stage (AJCC 7^th^ ed)	
Visceral pleural invasion	155 (35.22)	Premalignant lesion	1 (0.23)
Angiolymphatic invasion	123 (27.95)	1a	148 (33.64)
Tumor necrosis^3^	158 (37.80)	1b	141 (32.05)
Lymphocyte infiltrates^4^		2a	49 (11.14)
Minimal +Mild	267 (64.96)	2b	32 (7.27)
Moderate + Marked	144 (35.04)	3a	69 (15.68)

^1^ 3 patients had endobronchial lesion, 1 patient had central tumor necrosis

^2^ Vessel injury: 10 (2 pulmonary vein; 8 pulmonary artery); Resectability evaluation: 17; Airway reconstruction: 1

^3^ missing data in 22 patients

^4^ missing data in 29 patients

### Correlation between CT tumor component and pathologic findings

We analyzed the tumor size as determined by two different measurement modalities, including maximal diameter of the tumor in CT mediastinal window and the actual finding upon pathologic examination. There was significant correlation between these two values (Pearson correlation coefficient = 0.67, p-value < 0.001; Table 2) The mean sizes, as measured on CT and pathologic finding, were 2.97±1.85 and 2.99±1.63 cm, respectively. Furthermore, we re-evaluated the CT presentation and categorized patients into subgroups, including pure GGO, GGO predominant, solid predominant, and pure solid lesion, in order to analyze the correlation between tumor component and pathology findings. (Table 2) We found that most (75/77, 98.7%) pure GGO and GGO predominant lesions were adenocarcinoma. (p< 0.001) In addition, most (61/76, 80.2%) of these lesions were well differentiated. (p < 0.0001) Also, pure GGO lesions carried a lower risk of visceral pleura invasion (p < 0.0001), angiolymphatic invasion (p < 0.002), less tumor necrosis (p<0.0001), less lymphocyte infiltration (p = 0.0042) and tumor purity. (p <0.0001)

**Table 2:** Correlation between CT tumor component and pathologic findings.

Tumor size		Mean ± SD (cm)/(%)				Association
Pathology tumor size		2.99±1.63				p <0.001^6^
CT finding tumor size		2.97±1.85				
	Component^1^	Pure GGO CT ratio = 0	GGO dominant 0< CT ratio <0.5	Solid dominant 0.5 ≤ CT ratio <1	Pure solid CT ratio = 1	
Item						
Adenocarcinoma^1^		32 (97)	43 (97.7)	221 (89.6)	48 (53.3)	p<0.0001^7^
Well differentiated^2^		30 (93.9)	31 (70.5)	125 (48.3)	15 (19.7)	p <0.0001^8^
LN invasion status^1^ Metastatic N1 LN No.		0.03±0.17	0.25±0.87	0.39±0.98	0.75±1.60	p = 0.004^9^
Visceral pleural invasion^1^		0	17(38.6)	92 (34.3)	46 (50.1)	p<0.0001^8^
Angiolymphatic invasion^1^^,^^3^		1 (3.4)	8 (19.2)	83 (31)	31 (34.4)	p<0.002^7^
Tumor necrosis^4^		3 (9.38)	10 (23.81)	90 (35.43)	55 (63.22)	p<0.0001^10^
Lymphocyte infiltrates^5^ Minimal +Mild		25 (78.13)	(66.67)	166 (66.67)	45 (52.94)	p = 0.042^10^
Tumor purity		0.08±0.13	0.18±0.24	0.39±0.36	0.64±0.40	p<0.0001^10^

^1^ 4 patients were excluded from analysis (3 patients had endobronchial lesion, 1 patient had central tumor necrosis)

^2^ 29 patients were excluded from analysis (28 patients had no pathologic data for cell differentiation grade, 1 patient was confirmed atypical adenomatous hyperplasia)

^3^ 1 patient presenting as premalignant change without angiolymphatic invasion was excluded

^4^ missing data in 22 patients

^5^ missing data in 29 patients

^6^ Pearson correlation, coefficient = 0.67

^7^ chi-square test

^8^ Fisher exact test

^9^ Linear model

^10^ Cox regression

### Correlation between tumor component and lymph node metastatic pattern in different tumor sizes

Regarding patients’ survival, it is critical to assess the intrapulmonary (N1) and mediastinal (N2) nodal involvement. In this study, we found that pure GGO lesions had a lower risk of N1 lymph node metastasis (0.03 ± 0.17, p = 0.004, Linear model; Table 2). To clarify the real presentation, we further subdivided these four subgroups according to the tumor size as identified in chest CT mediastinal window view (Table 3). From the point of view of tumor size, among those with tumor size less than 1 cm, only one patient (1/26, 3.84%) was identified with mediastinal lymph node (N2) metastasis. From the point of view of tumor component, one patient (1/25, 4%) who presented with pure GGO lesions of size less than 2 cm had both intrapulmonary (N1) and mediastinal (N2) metastases. No lymph node metastasis (0/12) was identified among patients who presented as sub-centimeter pure GGO lesion. The lymph node metastasis rate of different tumor sizes was 3.8% (1/26) for tumor size less than 1 cm, 16.3% (15/92) for tumor size of 1 to 2 cm, 19.6% (30/153) for 2 to 3 cm, and 37% (61/165) for tumor size greater than 3 cm. The detailed lymph node metastasis pattern is shown in Table 3.

**Table 3:** Tumor component vs. lymph node metastatic pattern in different tumor sizes.

				Component^1^	Pure GGO C/T ratio = 0					GGO dominant 0< C/T ratio <0.5				Solid dominant 0.5 ≤ C/T ratio <1				Pure solid CT ratio = 1			
				Size	A^2^		B^2^	C^2^	D^2^	A^2^	B^2^	C^2^	D^2^	A^2^	B^2^	C^2^	D^2^	A^2^	B^2^	C^2^	D^2^
Lymph node status																					
No LN metastases					12 (100)		12 (92.3)	7 (100)	1 (100)	3 (100)	10 (100)	19 (79.2)	6 (85.7)	9 (90)	50 (79.4)	78 (81.4)	64 (64.6)	1 (100)	5 (83.3)	19 (76)	33 (56.9)
N1 metastases only					0 (0)		0 (0)	0 (0)	0 (0)	0 (0)	0 (0)	3 (12.5)	1 (14.3)	0 (0)	6 (9.5)	8 (8.2)	17 (17.2)	0 (0)	0 (0)	3 (12)	10 (17.2)
N2 metastases					0 (0)		0 (0)	0 (0)	0 (0)	0 (0)	0 (0)	1 (4.15)	0 (0)	1 (10)	3 (4.8)	5 (5.2)	7 (7.1)	0 (0)	0 (0)	0 (0)	5 (8.6)
N1/N2 metastases					0 (0)		1 (7.7)	0 (0)	0 (0)	0 (0)	0 (0)	1 (4.15)	0 (0)	0 (0)	4 (6.3)	6 (6.2)	11 (11.1)	0 (0)	1 (16.7)	3 (12)	10 (17.2)
No LN metastases					N1 LN metastases					N2 LN metastases				N1/N2 LN metastases				Total LN metastases			
A^2^	B^2^	C^2^	D^2^		A^2^	B^2^		C^2^	D^2^	A^2^	B^2^	C^2^	D^2^	A^2^	B^2^	C^2^	D^2^	A^2^	B^2^	C^2^	D^2^
25 (96.2)	77 (83.7)	123 (80.4)	104 (62)		0 (0)	6 (6.5)		14 (9.2)	28 (17)	1 (3.8)	3 (3.3)	6 (3.9)	12 (7.3)	0 (0)	6 (6.5)	10 (6.5)	21 (12.7)	1 (3.8)	15 (16.3)	30 (19.6)	61 (37)

^1^4 patients were excluded due to central necrosis (1) and endobronchial lesion (3)

^2^A: tumor <1 cm (N = 26); B: 1cm < size ≤ 2cm (N = 92); C: 2 cm< size ≤ 3 cm (N = 153); D: size > 3 cm (N = 165)

### Analysis for prognosis factor identification and patients’ survival

We also analyzed all clinicopathologic factors in order to identify the correlation to patients’ survival. Since pathologic findings were not available before surgery, we tried to identify the survival impact of clinical factors that could be known pre-operatively. In our study, we have identified GGO correlated pathologic findings (Table 2) and confirmed these by single variant analysis. (S1 and S2 Tables)

**Fig 4:**
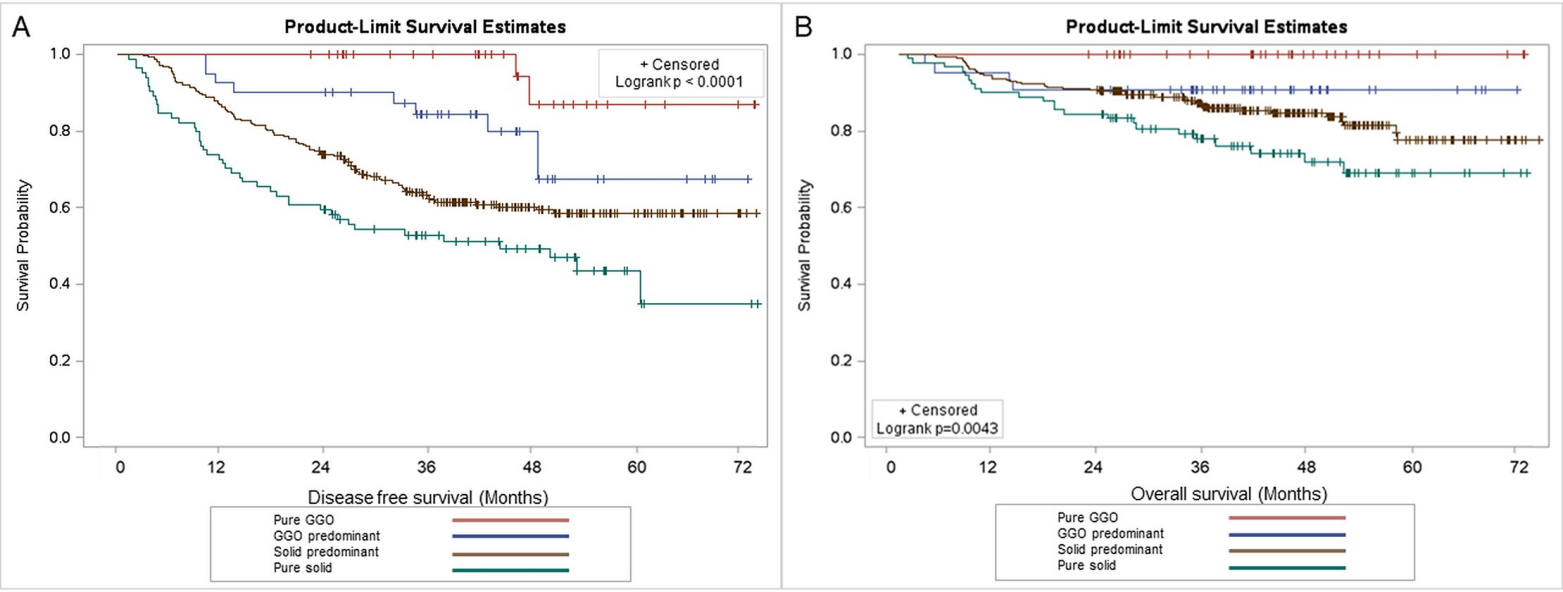
Disease free and overall survival for non small cell lung cancer patients for different C/T ratios. A. Disease free survival for non small cell lung cancer patients with different C/T ratio (p <0.0001) B. Overall survival for non small cell lung cancer patients with different C/T ratio (p = 0.0043).

Because GGO presentation was highly correlated to pathologic findings and all pathologic finding could not be known before operation, we used GGO to represent pathologic findings and compare with other clinical factors in a multivariant regression model. For multivariate analysis, we used GGO predominant lesion (C/T ratio < 0.5) to represent pathologic factors and included other clinical factors with a p-value less than 0.05 in the Cox proportional hazard model analysis. From this Cox proportional hazard model, we found that gender (p = 0.02) and GGO predominant tumor presentation (p = 0.0005) were correlated to DFS. (Table 4) In addition, a history of smoking (p = 0.03) and GGO predominant lesion (p = 0.04) were identified as correlated to OS. (Table 4) The 5-year DFS and OS of pure GGO lesion (C/T ratio = 0) were 87% and 100%, respectively. (Fig 4)

**Table 4:** Multivariable analysis for disease free and overall survival (cox proportion hazard model).

	Multivariable analysis for disease free survival (Cox proportion hazard model)				
	Statistical results	Parameter estimate	Standard Error	95% confidence interval	P value
_Variable_					
Gender		-0.38	0.17	0.49,0.95	0.02
CT tumor size		0.38	0.20	0.98,2.18	0.06
GGO		-1.07	0.31	0.19,0.62	0.000
Thoracotomy vs. VATS		0.29	0.28	0.78,2.31	0.28
	Multivariable analysis for Overall survival (Cox proportion hazard model)				
	Statistical results	Parameter estimate	Standard Error	95% confidence interval	P value
Variable					
Gender		-0.19	0.34	0.43,1.59	0.56
Smoke		0.68	0.32	1.06,3.66	0.03
CT tumor size		0.46	0.34	0.82,3.06	0.17
GGO		-1.05	0.52	0.13,0.98	0.04
Thoracotomy vs. VATS		0.62	0.33	0.98,3.56	0.06

## Discussion

Many studies have tried to analyze the correlation between image characteristics and pathologic findings of non small cell lung cancer. [23–25,32] The results have shown that the tumor solid component is correlated more with invasive pathologic characteristic and worse survival.[23,26]　However, most studies are based on thinner slice thickness[26,27, 35], and image characteristics change as slice thickness varies. There has been no consensus regarding CT slice thickness for non small cell lung cancer imaging. Pure GGO lesions revealed in 5 mm slice thickness may present as solid lesions in 1 mm slice thickness. Furthermore, no clinical data has demonstrated the correlation between actual lymph node involvement and tumor component where malignancy was identified.

In our study, the lymph node metastasis rate for different tumor sizes was 3.8% (1/26) for tumors less than 1 cm, 16.3% (15/92) for tumor size 1 to 2 cm, 19.6% (30/153) for 2 to 3 cm, and 37% (61/165) for tumors greater than 3 cm. In the clinical scenario, lymph node metastases occurred in both subcentimeter lesions and pure GGO lesions less than 2 cm, ie. the classification of T1a by the 7^th^ edition TNM staging system. Only patients presenting with pure GGO lesions less than 1 cm had no lymph node metastases. (0/12) Our findings enumerate the percentage of lymph node metastases in different scenarios. Furthermore, we propose that sub-centimeter pure GGO lesions with no lymph node metastases be managed as a specific population. For patients presenting as sub-centimeter pure GGO lesions without tissue proving, regular surveillance could suffice as a treatment option due to absence of lymph node metastases and low risk of visceral pleura and angiolymphatic invasion. However, the limited case number in our study warrants further investigation. For patients with other types of tumor components of size greater than 1 cm without tissue proving, prompt tissue proving and surgical resection after confirmed malignancy could improve patients' survival. In addition, we not only found the consolidation-tumor ratio revealed in CT with 5mm slice thickness to have good correlation to the pathology finding, but also to have good survival prediction power. Regular surveillance may be adequate for pure GGO lesions, given the excellent 5-year survival. Aggressive management is recommended for mixed GGO and solid lesions. This information is useful not only for physicians who deal with patients with lung nodules without tissue proof, but also can provide quantified survival data to help patients and their families with difficult decisions.

Despite our positive findings, some limitations remain. First, this is a retrospective study of medium size, which prohibits further detailed analysis. However, our study not only closes the gap between image and pathology but also analyzes the survival impact of consolidation tumor ratio and clinical factors. Second, there were variations between surgeons, physicians, and radiologists. We carefully chose the target image to minimize the inter-observation bias and had all the specialists evaluate each target image. We chose tumor size and consolidation-tumor ratio instead of other CT characteristics because the measurements are easily reproducible. Third, our results cannot be applied to patients presenting as endobronchial lesion with obstructive pneumonia or those presenting with cystic lesion related to central necrosis because it is difficult to clarify the tumor border in the CT image. Further investigation is warranted where better image tools are available. Fourth, the image characteristics of the mediastinal lymph node were not included in this study. There were no clear, definite criteria that could be utilized for lymph node involvement status.[36] The only way to confirm lymph node involvement status is to obtain tissue proof by invasive procedure, such as endobronchial ultrasonographic or mediastinoscopic biopsy.[37,38] Although limitations remain, our results reveal that tumor size and consolidation-tumor ratio are sufficient to stratify patients’ survival and are useful for doctors and patients to make decisions. For clinicians, our result could provide more information to support the clinical impression to plan a precise treatment program and avoid unnecessary operations. For patients and their families, our results can not only ameliorate psychological stress, but also provide definite quantified survival impact if malignancy is highly suspected. This can help them to make decisions more easily and confidently.

## Conclusion

No lymph node metastasis was identified in sub-centimeter pure GGO lesion. The consolidation tumor ratio could be used to differentiate patients’ disease and overall survival, while excellent 5-year overall survival was identified in pure GGO lesion.

## Supporting information

S1 Table Single variable analysis for disease free survival. (DOCX)

S2 Table Single variable analysis of overall survival. (DOCX)
