# Correction: Correlation between image characteristics and pathologic findings in non small cell lung cancer patients after anatomic resection

**DOI:** 10.1371/journal.pone.0212461

**Published:** 2019-02-12

**Authors:** Jui-Ying Fu, Yung-Liang Wan, Tzu-Yen Huang, Ching-Feng Wu, Yun-Hen Liu, Ming-Ju Hsieh, Yi-Cheng Wu, Ching-Yang Wu

There is an error in the Materials and methods section under the sub-heading “Patient selection and data collection.” In the last sentence of the first paragraph, the IRB number is listed incorrectly. The correct grant number is: 103-5631B.
