# Supplementary material for: Correlation between image characteristics and pathologic findings in non small cell lung cancer patients after anatomic resection
Source: PLoS One. 2018 Oct 31;13(10):e0206386. doi: 10.1371/journal.pone.0206386 (PMC6209293; doi:10.1371/journal.pone.0206386)
Supplement: S1 Table — (DOCX) [file pone.0206386.s001.docx]

S1 Table Single variable analysis for disease free survival

| _Variable¤_ ^Statistical results¤^ | Parameter estimate¤ | Standard Error¤ | 95% confidence interval¤ | P value¤ |
| --- | --- | --- | --- | --- |
| Patient factor¤  Age   ( ≥60 years vs. <60 years)¤  Gender (Female vs. Male)¤  Smoking ( Yes vs No)¤ | ¤  0.22¤  -0.41¤  0.31¤ | ¤  0.17¤  0.17¤  0.18¤ | ¤  0.89,1.74¤  0.45,0.93¤  0.95,1.95¤ | ¤  0.20¤  0.016¤  0.09¤ |
| Image factor¤  GGO (consolidation tumor ratio <50% vs.≥ 50%)¤  CT tumor size ( < 2cm vs. ≥ 2cm)¤ | ¤  -1.17¤  0.51¤ | ¤  0.31¤  0.22¤ | ¤  0.17,0.57¤  1.09,2.55¤ | ¤  0.002¤  0.02¤ |
| Surgery factor¤  Thoracotomy vs. VATS¤ | ¤  0.53¤ | ¤  0.26¤ | ¤  1.03,2.84¤ | ¤  0.04¤ |
| Pathologic factor   Pathology tumor size (≥2 cm vs. <2 cm)¤   Cell type (Adenocarcinoma vs. non adenocarcinoma)¤   Well Differentiation grade¤   Visceral pleural invasion (Yes vs. No)¤   Angiolymphatic invasion (Yes vs. No)¤   Tumor purity¤   Lymphocyte infiltrates¤  (Mild + Minimal vs. Moderate +Marked)¤   Tumor necrosis (Yes vs No)¤   Stage ( 1a, 1b, 2a, 2b, 3a)¤      1b vs. 1a¤      2a vs. 1a¤      2b vs. 1a¤      3a vs. 1a¤ | ¤  0.96¤  0.14¤  -0.76¤  0.60¤  1.09¤  -0.05¤  -0.25¤  ¤  0.77¤  ¤  0.85¤  1.29¤  1.15¤  1.92¤ | ¤  0.26¤  0.25¤  0.18¤  0.17¤  0.17¤  0.02¤  0.18¤  ¤  0.17¤  ¤  0.28¤  0.33¤  0.38¤  0.29¤ | ¤  1.57,4.35¤  0.71,1.86¤  0.33,0.66¤  1.30,2.56¤  2.11,4.17¤  0.92,0.99¤  0.54,1.09¤  ¤  1.53,3.02¤  ¤  1.36,4.07¤  1.91,6.87¤  1.51,6.63¤  3.85,12.07¤ | ¤  0.002¤  0.58¤  <0.0001¤  0.0005¤  <0.0001¤  0.014¤  0.15¤  ¤  <0.0001¤  ¤  0.002¤  <0.0001¤  0.002¤  <0.0001¤ |
| Therapeutic factor¤    Post-op adjuvant therapy (Y versus N)¤ | ¤  0.93¤ | ¤ 0.17¤ | ¤ 1.80,3.57¤ | ¤ <0.0001¤ |
