# Supplementary material for: Correlation between image characteristics and pathologic findings in non small cell lung cancer patients after anatomic resection
Source: PLoS One. 2018 Oct 31;13(10):e0206386. doi: 10.1371/journal.pone.0206386 (PMC6209293; doi:10.1371/journal.pone.0206386)
Supplement: S2 Table — (DOCX) [file pone.0206386.s002.docx]

S2 Table Single variable analysis of overall survival

| _Variable¤_ ^Statistical results¤^ | Parameter estimate¤ | Standard Error¤ | 95% confidence interval¤ | P value¤ |
| --- | --- | --- | --- | --- |
| Patient factor¤  Age   ( ≥60 years vs. <60 years)¤  Gender (Female vs. Male)¤  Smoking ( Yes vs No)¤ | ¤  0.49¤  -.097¤  1.08¤ | ¤  0.25¤  0.25¤  0.24¤ | ¤  1.01, 2.67¤  0.23, 0.62¤  1.84, 4.71¤ | ¤  0.044¤  <0.0001¤  <0.0001¤ |
| Image factor¤  GGO(consolidation tumor ratio <50% vs.≥ 50%)¤  CT tumor size ( < 2cm vs. ≥ 2cm)¤ | ¤  -1.29¤  0.93¤ | ¤  0.51¤  0.36¤ | ¤  0.10,0.76¤  1.26,5.09¤ | ¤  0.012¤  0.01¤ |
| Surgery factor¤  Thoracotomy vs. VATS¤ | ¤  1.06¤ | ¤  0.31¤ | ¤  1.58, 5.26¤ | ¤  0.0006¤ |
| Pathologic factor¤   Pathology tumor size (≥2 cm vs. <2 cm)¤   Cell type ( Adenocarcinoma vs.non adenocarcinoma)¤   Well Differentiation grade¤   Visceral pleural invasion (Yes vs. No)¤   Angiolymphatic invasion (Yes vs. No)¤   Tumor purity¤   Lymphocyte infiltrates¤  (Mild + Minimal vs. Moderate +Marked)¤   Tumor necrosis (Yes vs No)¤   Stage ( 1a, 1b, 2a, 2b, 3a)¤      1b vs. 1a¤      2a vs. 1a¤      2b vs. 1a¤      3a vs. 1a¤ | ¤  1.13¤  -0.47¤  -0.54¤  1.17¤  1.05¤  -0.14¤  -0.26¤  ¤  -0.27¤  ¤  1.39¤  1.57¤  1.22¤  2.05¤ | ¤  0.39¤  0.26¤  0.27¤  0.25¤  0.24¤  0.05¤  0.25¤  ¤  0.25¤  ¤  0.42¤  0.49¤  0.59¤  0.44¤ | ¤  1.42,6.74¤  0.37,1.05¤  0.34,0.99¤  1.96,5.31¤  1.78,4.61¤  0.79,0.96¤  0.47,1.27¤  ¤  0.46,1.25¤  ¤  1.76,9.30¤  1.82,12.58¤  1.08,10.70¤  3.31,18.34¤ | ¤  0.004¤  0.08¤  0.048¤  <0.0001¤  <0.0001¤  0.004¤  0.30¤  ¤  0.29¤  ¤  0.001¤  0.002¤  0.036¤  <0.0001¤ |
| Therapeutic factor¤    Post-op adjuvant therapy (Y versus N)¤ | ¤  0.49¤ | ¤ 0.25¤ | ¤ 0.99,2.65¤ | ¤ 0.05¤ |
